# Effects of Different Teaching Approaches on Proxy Measures of Physical Fitness of Italian Kindergarten Children

**DOI:** 10.3390/ijerph20105792

**Published:** 2023-05-11

**Authors:** Patrizia Tortella, Antonella Quinto, Guido Francesco Fumagalli, Mario Lipoma, David Stodden, Francesco Sgrò

**Affiliations:** 1 Faculty of Human and Society Sciences, University of Enna “Kore”, 94100 Enna, Italyantonella.quinto@unikore.it (A.Q.);; 2 Research Center on Child Motor Development, Department of Diagnostics and Public Health, University of Verona, 37129 Verona, Italy; 3 Department of Physical Education, University of South Carolina, Columbia, SC 29208, USA

**Keywords:** physical fitness index, playground, free play, structured activity

## Abstract

Developing physical fitness (PF) behaviors early in life enhances physical development and facilitates sustained participation in physical activity and sports across childhood. This study addressed the effect of different teaching approaches on precursors of PF in kindergarten children. A total of 178 children (5.45 ± 0.40 years, female = 92) from 11 classes were organized into three groups. Group 1 (structured activity + free play) and Group 2 (only free play) attended the same playground (PrimoSport0246) for one hour/week for 10 weeks. Group 3 (structured activity + free play in kindergarten) followed their standard physical education curriculum at school. PF tests (standing long jump, medicine ball throw, and 20 m running speed) were administered pre- and post-intervention. Factorial ANOVA was implemented using a percentage change in PF performance (PFC) as the dependent variable, and teaching approaches, gender, and age as factors. Group 1 demonstrated significant improvements in fitness performance compared with Groups 2 and 3. Moderate to large effect sizes (Cohen’s *d* range: 0.68–1.40) were noted in both males and females. Six-year-old demonstrated the greatest improvement in composite PFC compared to Groups 2 and 3. A structured teaching plan, even with a limited dose of once per week, supported the development of PF attributes in kindergarteners.

## 1. Introduction

Childhood is an important period for physical development by means of adequate learning experiences and opportunities to develop multiple attributes of physical functioning. “Physical fitness is the capacity to perform physical activity and makes reference to a full range of physiological and psychological qualities” [1]. Historically, two categories of measurable components that contribute to physical fitness are “health-related fitness” and “skill-related fitness” [2]. Health-related fitness components are body composition, cardiorespiratory endurance, flexibility, muscular endurance, power, and strength. Skill-related fitness has generally included assessments of balance, coordination, speed, reaction time, and agility. While these two components have generally been separated from a conceptual standpoint, assessments to measure the two constructs are historically problematic from an application perspective, as some assessments have been interchangeably used in research (e.g., standing long jump and running speed). The integrative nature of physical development in multiple domains, whether it is called “health-related” or “skill-related”, is particularly evident when assessing physical fitness in young children. For example, projecting/striking an object (e.g., balls of different sizes and masses) or projecting the body (e.g., jumping or hopping) with high levels of effort requires not only complex multijoint coordination, balance, and agility, but also the optimal application of force per unit of time (i.e., strength and power) [3,4,5]. From a developmental perspective, health-related or skill-related attributes of physical performance in early childhood are not mutually exclusive as children learn to manipulate their bodies in space and various objects of different sizes and masses as they grow. In addition, the synergistic integration of biological growth and neuromuscular maturation with experience-based, effortful practice and repetition of various movements leads to an increased capacity to generate force via inter- and intra-muscular coordination and control, muscular endurance, and cardiorespiratory endurance. Thus, a broad foundation of physical activity opportunities facilitates the conjoint development of functional capabilities from both health and skillfulness perspectives. From a long-term perspective, providing developmentally appropriate learning opportunities facilitates a foundation for continued engagement in a variety of health-enhancing physical activities [6,7] and sports [8] from young childhood to adult phases of life.

Since physical development habits and behavior patterns are established from an early age, it is important that teachers in kindergarten promote the exploration of movement through a variety of activities so children can discover many ways to enjoy movement [9] and reap its multiple benefits [10]. Indeed, there is strong evidence that a physically active lifestyle and adequate levels of physical fitness from a young age help to improve health in multiple domains [11,12]. This phenomenon can be explained through the concomitant development of health- and skill-related physical capabilities [4,13,14,15,16,17]. For this reason, it is essential to follow the World Health Organization’s (WHO) global recommendations on physical activity for health (2020) to deliver at least 60 min of moderate- to vigorous-intensity physical activities each day for children 5–17 years old to improve their cardiorespiratory and muscular fitness and bone health [18].

Unfortunately, Żegleń et al. (2020) demonstrated negative changes in most physical fitness levels administered in young children (i.e., 3–7 years old) across different generation cohorts [19]. As physical fitness trajectories follow a developmental trajectory, these declines may manifest across childhood and adolescence [16]. Ortega and colleagues (2023) also demonstrated low muscular strength levels in adolescents from countries in southern Europe compared to the central and northern countries of Europe [20]. These trends have potential repercussions on the health of today’s children and, consequently, on tomorrow’s adults. Of course, one of the most important contexts for promoting physical fitness is in schools. However, generalist teachers who work in kindergarten often complain that they are not properly prepared to promote physical fitness development within the physical education teaching-learning process [21,22]

In agreement with Popeska and colleagues (2020), we believe quality PE experiences should be mandatory from kindergarten to help the development of physical fitness from an early age [23]. In addition, it is necessary to develop PE curricular strategies which account for the environmental characteristics of particular situations that can most optimally contribute to the development of physical fitness [24]. In this sense, a faithful ally of health-enhancing physical fitness activities in kindergartners is the playground. Playing games and individual play in outdoor spaces with equipment that inherently promotes improvement in multiple domains of fitness is possible in most playgrounds, specifically, as there are usually many different types of activities that can be pursued in playgrounds. However, facilitating consistent activity for all children speaks to the importance of delineating between teacher-facilitated engagement in health-enhancing physical activities and free play. To the best of our knowledge, studies focused on the effect of the use of different teaching approaches, which accounts for different methods and learning environments, to support the development of PF in kindergartners are rare.

Commonly, teachers promote different types of structured activities in kindergartners [25]: basic movement activities, rhythmic activities, physical games, and sports. Structured physical activities can facilitate the development of physical fitness components [26,27], even if an understanding of the impact of various activities is lacking. Additionally, the impact of participating in different environments, specifically incorporating playground equipment of various kinds, general education, or free play on PF development in early childhood is not well understood.

The playground is a natural space where multiple affordances offer several possibilities for various physical development opportunities. A playground can be defined as an “active” place that can support the development of physical and cognitive skills where structured activities are delivered by an expert who supports sustained participation in various ways (i.e., scaffolding) [28,29,30,31].

On that basis, the main aim of this study was to assess the effects of different teaching methods and learning contexts on precursors of physical fitness in kindergarten children. Specifically, we hypothesized that teaching approaches based on structured physical activity and free play delivered in the playground learning context would lead to significantly greater PF improvements in kindergarten children compared to PA time promoted in school.

## 2. Materials and Methods

### 2.1. Design

A quasi-experimental study, with convenience cluster sampling and a non-equivalent control group, pre- and post-test design was used in this study. Due to the educational nature of this study, an in-tact class was used as the criterion for the cluster sampling, and this permits us also to support the ecological perspective required in educational-based research.

### 2.2. Participants and Procedures

A total of 182 children from 11 classes of 7 kindergartens in a region in northeastern Italy were chosen based on their similarity in socioeconomic status and ethnic origin of the children’s families and whether they were able to visit the playground. The playground is located within the “Cittadella dello Sport-La Ghirada”, in Treviso, Italy and was designed to promote motor development in children aged 0–6 years [28]. Experts from the local branch of the Italian National Olympic Committee of Treviso collaborated tutoring children at the park, as described in the next Section 2.4. Children participated in a 10-week study meeting once a week. The selected classes were randomly assigned to one group according to three different physical education approaches. Group 1: 4 classes, 73 children (mean age 5.41 ± 0.41, female = 37) participated in structured activity + free play at the playground; Group 2: 4 classes, 49 children (mean age 5.48 ± 0.39, female = 31) practiced only free play in the playground; Group 3: 3 classes, 60 children (mean age: 5.43 ± 0.40, female = 26) participated in structured activity + free play in their kindergarten environment.

Groups 1 and 2 carried out the activity on the playground in the permanent presence of members of the research staff who were specifically trained in providing structured activity in the playground. Group 3 carried out all activities in kindergarten in the presence of the classroom teacher only.

### 2.3. Environment and Design of the Study

The playground used by Groups 1 and 2 had a rectangular area of 2500 square meters (about 30 × 85 m). The ground was grassy and flat and was surrounded on one side by a hill. There were normal playground equipment grouped into the following areas of functioning: manual dexterity (different types of climbing equipment), balance (different types of balance equipment of different heights), and mobility (to promote locomotor skills on different surfaces uphill and downhill). In addition, there was other equipment, such as slides, swings, a hill space, and trees, where children could participate in a variety of locomotor and climbing activities. Group 3 kindergarten had a gymnasium (100–200 square meters) and outdoor play area (800–1000 square meters) where they could participate in a variety of locomotor activities at the teacher’s discretion. Typical playground equipment (e.g., slides, swings, climbing equipment, balance equipment, etc.) were available in the school’s outdoor space. In the gym, children could use hoops, balls, sticks, balance tools, and other informal materials (e.g., newspaper, cloths, and cardboard boxes).

This study was carried out in the years 2016–2017. Permission from the participating schools was obtained, and only children with informed consent of parents/or guardians and permission from schools participated in this research. This project was recognized as conforming to the Declaration of Helsinki and approved by the Scientific Committee of the nonprofit association “Laboratorio 0246”. Laboratorio 0246 owned the site (i.e., playground) where the research took place and helped organize the activity with schools.

### 2.4. Teaching Approaches

**Figure 1:**
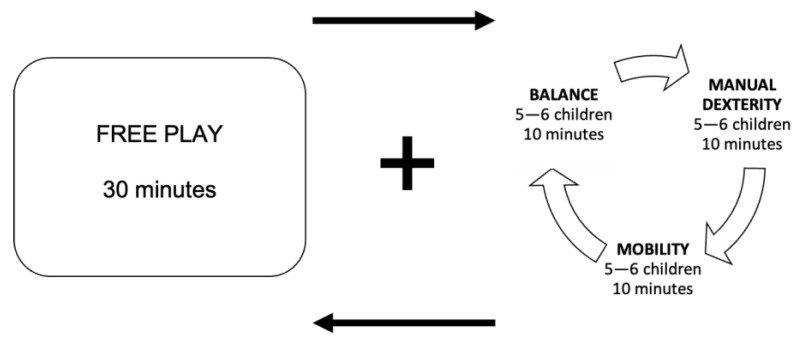
Organization of the activities of Group 1.

Group 1 (structured activity + free play at the playground). The children arrived at the playground and were divided into two groups. Group A carried out 30 min of structured activity in the three areas of the park (i.e., dexterity, mobility, and balance), while at the same time, Group B carried out 30 min of free play in the remaining spaces of the playground. After 30 min, the groups reversed activities. At the next meeting (i.e., next week), Group A started with free-play activities, while Group B began with structured activities, and this alternation was proposed every week. In order for the children to be better supported during the structured activity, they were divided into three small groups (5–6 children). Each group positioned itself in an area where they carried out circuit activities on the tools for 10 min and then rotated in the next area for another 10 min until the end of the 30 min, as described in Figure 1. In each area, there was an expert who provided guidance, support, and feedback to the children. A general coordinator of the activities checked the timing and gave the signals of zone change.

Group 2 (free play at the playground) had 60 min of free play, and the children could do whatever they wanted inside the park spaces. Kindergarten teachers accompanied the children to the playground and stayed inside the park but did not interact with the children. They were asked by researchers not to interfere in the children’s activity.

Group 3 (structured activity + free play in kindergarten) carried out structured activities and free play in their normal kindergarten environment. The teacher started the activities with free play, went ahead with the structured activity, and in the following week, they changed the order of these activities. This alternation was proposed every week. The selected schools had generalist teachers with degrees in physical education. In Italy, a specific degree in Education is needed to become a preschool teacher. Some teachers also had a degree in Physical Activity and Sport Sciences. The teachers promoted both structured activities and free play that were in line with the learning goals specified in the National Guidelines for the School Curricula (basic Italian normative text for kindergarten curricula). During free play, the children were allowed to participate in various activities in any way they wanted. Structured activity was organized through games and circuits, with the use of formal (balls, hoops, sticks, and balance axes) and informal materials (newspapers, fabrics, etc.).

All involvement monitoring activities were carried out in the same way for all three groups. Specifically, we used a “fidelity check” to verify attendance, timing, and organization of structured activities and free play, teachers’ roles, scaffolding, and feedback, children’s attendance, and children’s involvement in both park and kindergarten. In detail, a fidelity control sheet was built in agreement with previous studies [32,33] and was used to account for 30 items that were able to assess the above-cited activities’ characteristics. Each item was scored with two values: 1 if the assessed characteristic was verified and 0 if otherwise. A percentage score was obtained via initially summing all the items’ scores; then, this result was divided by 30 and multiplied by 100. We collected video for 40% of the sessions for each approach; two researchers assessed each video by means of the fidelity control sheet and found a 100% concordance of their evaluations on all videos. Concerning the validity of each approach, the researchers found a concordance of 98% at the playground and 80% related to the activities developed by the teachers and children in kindergarten. Free play was also observed to verify that teachers and experts did not influence the activities and did not lend support and feedback.

### 2.5. Assessment of Physical Fitness

The following three tests were selected in this study: medicine ball throw, standing broad jump, and running 20 m sprint. These tests were used for assessing precursors of musculoskeletal fitness components [34,35,36,37,38,39,40]. The tests used in this study have been used in several previous studies related to the assessment of lifespan PF (i.e., from preschool to adult time) [34,35,36,37,38,39,40] and also are included in several specific batteries, such as PreFIT [35] and OSF [39].

Medicine ball throw: The child starts with the feet parallel to each other and shoulder-width apart, with the ball (diameter 20 cm, weight 1 kg; Giodicart, Italy, Cod. 5401, type Trial) held against the chest. The child pushes the medicine ball horizontally with two hands as far as possible. The distance from the starting position to where the ball lands is measured in centimeters. The better of two attempts was used for analysis.

Standing broad jump: The starting position is with the child standing with feet parallel and shoulder-width behind the starting line. The child jumps as far as possible. The distance from the starting line to the back of the closest heel at landing is measured in centimeters. The better of two attempts was used for analysis.

Running 20 m as fast as possible: The child starts from a standing position behind the starting line and runs 20 m as fast as possible. The time in seconds was used for analysis.

### 2.6. Data Analysis

Data from the three tests were preliminarily checked for accuracy, missing values, equality of variances, and univariate outliers. Participant data were included if a child was present for at least 85% of the activity sessions and was able to participate in all requisite activities. Then, for each fitness test and participant, the percentage change in performance from pre-test to post-test was estimated according to the following formula:

Δ__physical fitness test_ (%) = [(score_post)/(score_pre) × 100] − 100.

This way, we obtained scores that could be combined together to estimate a global Physical Fitness percentage Change (%PFC). The negative values noted in the running test (i.e., decreased time = improvement) were reverse scored to align values for the summed %PFC change score. The following formula was used for estimating a global percentage change in the Physical Fitness level of each participant:

%PFC = Δ_medicine ball throw_ + Δ_standing broad jump_ + Δ_20-meter run._

A Factorial Analysis of Variance (Factorial ANOVA) was performed to understand the effect of teaching approaches, sex, age, and their interactions on the global percentage change in physical fitness performance. Main effects and interactions and simple main effects were estimated, and subsequent post hoc tests were performed using Holm corrections. The mean differences (MD) and 95% confidence interval (CI) were reported for the between-group analysis. Effect sizes (η^2^) were reported for main effect model and interpreted with the following cut-offs: 0.01 = small, 0.06 = medium, and 0.13 = large [3]. Cohen’s *d* (ES) was used for post hoc analysis and interpreted with the following cut-offs: 0.2 = small; 0.5 = medium; 0.8 = large [3]. The analyses were performed using JASP (version 0.16.4 for Apple Silicon), and the alpha test was set to 0.05.

## 3. Results

Data screening revealed that four participants resulted as univariate outliers. Because no other violations were verified, the data of these children were removed from the dataset, and parametric analyses were carried out on %PFC by considering a total sample of 178 (5.58 ± 0.36 years). Table 1 shows the descriptive statistics of participants’ performance on the different tests.

In Group 1, 5-year-old females’ %PFC was 4.71 ± 13.58, while males’ %PFC was 5.13 ± 10.49. For 6-year-old, the %PFC for females was 7.76 ± 6.68, while for males, it was 5.49 ± 5.58. In Group 2, %PFC mean and standard deviation of 5-year-old females resulted in −4.18 ± 16.37, while for males, it was 6.49 ± 14.28; concerning %PFC mean and standard deviation of 6-year-old females resulted in −5.68 ± 15.83, while for males, it was −5.17 ± 9.78. Finally, in Group 3, %PFC mean and standard deviation of 5-year-old females resulted in 1.05 ± 8.02, while for males, it was 1.62 ± 11.08; concerning %PFC mean and standard deviation of 6-year-old females resulted in −8.20 ± 15.30, while for males, it was −2.52 ± 8.06.

**Figure 2:**
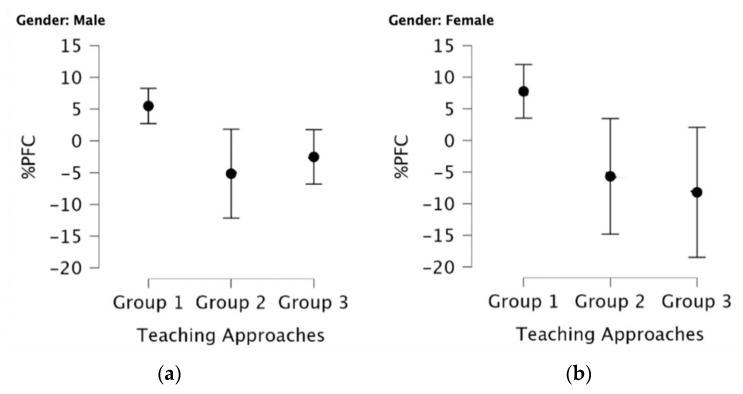
(**a**) Variation of %PFC for six-year-old males across the approaches (*F* = 3.31, *p* = 0.04); (**b**) Variation of %PFC for six-year-old females across the approaches (*F* = 6.39, *p* = 0.002). Note: Group 1 = structured activity + free play in the playground; Group 2 = free play in the playground; Group 3 = structured activity in kindergarten.

Factorial ANOVA revealed a significant main effect for the teaching approach (*F*(2,166) = 10.11, *p* < 0.0001, η^2^ = 0.10). Simple main effect analyses across teaching approaches at gender levels showed significant differences in males (*F* = 3.34, *p* = 0.03) and females (*F* = 8.33, *p* < 0.001). Simple main effect across approaches with the age-group factor revealed significant differences only for the six-year-old (*F* = 9.56, *p* < 0.001). Furthermore, simple main effects across approaches were found for the interaction between gender and age (i.e., six years), as shown in Figure 2.

Because the teaching approach is a three-level factor, post hoc analyses were carried out to understand the meaning of main and simple main effect results. Post hoc analyses showed that higher %PFC was demonstrated for participants who followed the activities of Group 1 in comparison with Group 2 (MD = 7.91, 95%CI = [2.47, 13.14], ES = 0.68, 95%CI = [0.20, 1.15], *p* = 0.002) and with Group 3 (MD = 8.60, 95%CI = [3.61, 13.58], ES = 0.73, 95%CI = [0.30, 1.17], *p* < 0.001). The differences were significant and demonstrated medium effect sizes in both cases but were highest for the comparison between Group 1 vs. 3.

Significant differences were demonstrated for the same comparisons when gender effect was accounted for. Specifically, analyses revealed a significant difference between the %PFC of females in Group 1 versus females in Group 2 (MD = 11.16, 95%CI = [2.59, 19.73], ES = 0.95, 95%CI = [0.20, 1.71], *p* = 0.004) and versus females in Group 3 (MD = 9.81, 95%CI = [0.78, 18.84], ES= 0.84, 95%CI = [0.04, 1.63], *p* = 0.03). In addition, a significantly higher %PFC was found for males who were in Group 1 versus males in Group 3 (MD = 7.36, 95%CI = [1.71, 13.00], ES= 0.75, 95%CI= [0.16, 1.34], *p* = 0.008).

Finally, significant differences were found for six-year-old males in Group 1 versus their peers in Group 2 (MD = 10.66, 95%CI = [3.73, 17.94], ES = 1.40, 95%CI = [0.42, 2.38], *p* = 0.003), and in Group 3 (MD = 8.01, 95%CI = [1.67, 14.36], ES= 1.05, 95%CI = [0.19, 1.92], *p* = 0.011). %PFC of six-year-old females in Group 1 demonstrated significantly higher %PFC change compared to Group 2 (MD = 13.43, 95%CI = [0.53, 26.34], ES = 1.04, 95%CI = [0.01, 1.99], *p* = 0.04) and of Group 3 (MD = 15.96, 95%CI = [2.27, 29.65], ES = 1.19, 95%CI = [0.14, 2.24], *p* = 0.02).

In essence, the teaching method for Group 1 (structured activity + free play) produced significant and medium-to-large improvements in the participant’s %PFC in comparison with the other groups, specifically in the six-year-old participants.

## 4. Discussion

This present study addressed the effect of using different teaching approaches to support the development of the precursors of physical fitness in kindergarten children.

The use of structured activities combined with free play, delivered in a playground, promoted improvements in performance levels of the composite physical fitness score of the current participants, even with the limited dose of only one time/week. Although there was a net positive change in fitness performance in both 5-year-old boys and girls in the structured/free-play group, greater performance increases were demonstrated in the 6-year-old boys and girls.

The positive changes in performance in the structured/free-play group were in contrast to the general lack of performance improvement or even decreased mean performance in PFC in children in the other two groups across 10 weeks. The increase in mean performance in the structured/free-play group, combined with a decrease or lack of improvement in the other two groups partially explains the medium to large effect sizes noted in the analyses. In this respect, the provided hypothesis was verified. As research on the development of precursors to PF in kindergarten children is rare, this current study provides a novel finding on a critical topic and provides several implications for the health and well-being of the future generation.

Teaching approaches resulted in the only significant main factor, and the relative effect was medium (η^2^ = 0.10). While children participated in the intervention only one time/week, this result signified that the significant changes in PF for Group 1 were impacted by the structured activities that were promoted. This outcome aligns with previous evidence about the relevant role of promoting adequate structured approaches for kindergartens [27]. A possible explanation for these results may be the lack of using a structured teaching protocol in Groups 2 and 3. This suggests that, in addition to the learning environment, the use of structured teaching methods that facilitates consistent engagement in a variety of fitness-enhancing activities promotes changes in PF. In this respect, it should be noted that the Italian National Guidelines prescribe learning outcomes, but the guidelines document does not offer specific strategies or teaching methods to facilitate the achievement of PF. As a consequence, the implementation of teaching approaches is often dependent on the teachers’ experience in promoting development [41,42]. For this reason, the current findings corroborate the ideas of Ward (2013), who suggested that without a deep understanding of the content to be taught, teachers are not able to achieve significant results in physical education [43,44]. This is also in agreement with evidence provided by Mak, Chan, and Capio (2021), who noted that it is essential to take into account the challenges and barriers that teachers may encounter in implementing physical activity and fitness promotion programs in early childhood [22]. One of the barriers, as shown by another study, is the absence of a standardized physical education curriculum for kindergarten [44]. For this reason, teachers must be informed about what basic contents should be covered in physical education lessons and how these contents have to be met as a prerequisite for achieving the necessary competence to build quality and effective learning sessions [44].

At the same time, this current study accounts for the use of different learning environments to promote the development of PF attributes in early childhood. Although it has been shown that well-structured programs improve the PF of children [26,27], only a few previous studies addressed the effectiveness of the playground as a context for promoting structured learning opportunities aimed at the development of PF in young children. In this respect, the current results suggest that the appropriate use of a playground environment for promoting physical education activities is able to stimulate PF improvements in kindergartners. Accordingly, some previous studies showed playgrounds are positively associated with children’s physical activity (PA) [24,38,45,46]. In particular, the level of kindergarteners’ physical activity is directly associated with the availability of equipment on a playground [45]. Furthermore, one study suggested that the development of both health-related fitness (muscle strength and aerobic capacity) and skill-related fitness (power, agility, and speed) may make it easier for kindergarten children to sustain their engagement in physical activities as they can be more successful in the activities in which they are engaging [6].

Subsequent simple main effect analysis revealed that the changes in PF due to the different teaching approaches were demonstrated both for males and females, regardless of participants’ age. Males in Group 1 obtained a significant and medium improvement in their PF of 7.4% compared to the males in Group 3. Females in Group 1 demonstrated a large difference in their PF (11.2%) compared to the females in Group 2 and a 9.8% difference compared to their peers in Group 3. A previous study provides evidence that children are differently engaged in the playground zones according to their gender. Boys were more likely to perform more vigorous PA in zones without equipment, and girls were more likely to use zones with equipment [47]. In this respect, Stellino and Sinclair (2014) demonstrated that girls participated in a higher number of typical playground activities compared with boys who participate in more sport-related activities [48]. Results of this study suggest that a mixed method, such as the one used in Group 1, could specifically stimulate females’ improvement in PF by means of a structured phase, and males may take greater advantage of the free play opportunities. However, this phenomenon needs to be further investigated to be confirmed.

The current results also revealed greater changes in PF in six-year-old participants, regardless of their gender. Specifically, large and significant improvements were found in males’ PF levels in Group 1 versus their peers in Group 2 (10.7%) and Group 3 (8.01%). Similar trends were noted in six-year-old females in Group 1 versus their peers in Group 2 (13.4%) and Group 3 (16.0%). Because specific evidence on the effect of different environments combined with different teaching methods in kindergartners is rare, we wondered which aspect of the protocol might be most functional, from a conceptual perspective, for explaining the improvements of six-year-old participants involved in the structured activities at the playground. In this respect, Heft (1988) affirmed that objects in the environment have an evolutionary dimension [49]. In particular, the possibilities of action offered by an environmental change in relation to the age status of the person. For example, a gap in an enclosure may stimulate locomotion in a young child but not in an older one. Thus, some functional possibilities of an environment may exist in one moment of life yet be viewed differently at a later age, or vice versa. Similarly, as we grow older, new offerings may emerge as a result of experience and expand the person’s behavioral repertoire [49]. The six-year-old children of Group 1 may have engaged with the playground tools differently than the five-year-old children of the same group, stimulating the development of their PF. In addition, six-year-old children of Group 2 did not achieve these improvements, although they were left free to act. Another important aspect of these data is the fact that children did not demonstrate changes in PF with free play, even in a novel and affordance-rich environment. Honomichl and Zhe Chen (2012) show evidence that guidance should be strategically included in discovery learning (i.e., Group 1) such that children’s discovery of new action possibilities with playground tools is supported via the initial structured learning [50].

The results provided in this study have the following limitations. Different teachers promoted structured activities for participants in the classes of Group 3, so potential differences in teaching styles may have impacted the PF development in this group. However, the secondary analysis demonstrated no significant differences in PF changes between the children with different teachers.

## 5. Conclusions

This study provides evidence that the use of structured teaching and free play in the playground environment was a more effective strategy than either free play or the normal physical education developed in a school environment for improving precursors to physical fitness in this sample of kindergartners. Because studies on promoting PF in kindergartners are rare, especially in Italy, the results of this study extend the current literature and the current level of Italian evidence on this topic. This is critically important in Italy because, in 2022, PE in primary school has been assigned to experts with a master’s degree in physical activity and sport science fields. These experts will be involved with children in the first year of primary school and should understand that kindergartners’ PF is positively influenced by a mixed teaching strategy where it is important to introduce different forms of movement. In conclusion, the provided results represent a base for developing additional studies aimed to expand on these results.

## Figures and Tables

**Table 1:** Descriptive statistics (mean and standard deviation) of tests’ results according to groups.

	Medicine Ball Throw [cm]		Standing Broad Jump [cm]		Running 20 m [s]	
	Pre-Test	Post-Test	Pre-Test	Post-Test	Pre-Test	Post-Test
Group 1	203.85 ± 43.64	218.88 ± 50.00	92.21 ± 19.88	98.85 ± 21.84	6.21 ± 0.79	5.85 ± 0.78
Group 2	197.74 ± 53.80	211.57 ± 45.82	90.26 ± 20.82	95.96 ± 15.98	6.05 ± 1.20	6.14 ± 0.85
Group 3	194.77 ± 46.81	205.57 ± 48.49	89.03 ± 13.98	94.15 ± 15.02	6.03 ± 0.75	6.15 ± 0.79

Note: Data are presented as mean ± standard deviation. cm = centimeters; s = seconds.

## Data Availability

All relevant data are contained in this article. The dataset analyzed in this study is available from the corresponding author upon reasonable request.
